# Genome-wide prediction and identification of *cis*-natural antisense transcripts in *Arabidopsis thaliana*

**DOI:** 10.1186/gb-2005-6-4-r30

**Published:** 2005-03-15

**Authors:** Xiu-Jie Wang, Terry Gaasterland, Nam-Hai Chua

**Affiliations:** 1 Laboratory of Computational Genomics, The Rockefeller University, New York, NY 10021, USA; 2 Institute of Genetics and Developmental Biology, Chinese Academy of Sciences, Beijing 100101, China; 3 Scripps Institution of Oceanography, University of California San Diego, La Jolla, CA 92093, USA; 4 Laboratory of Plant Molecular Biology, The Rockefeller University, New York, NY 10021, USA

## Abstract

**Background:**

Natural antisense transcripts (NAT) are a class of endogenous coding or non-protein-coding RNAs with sequence complementarity to other transcripts. Several lines of evidence have shown that *cis*- and *trans*-NATs may participate in a broad range of gene regulatory events. Genome-wide identification of *cis*-NATs in human, mouse and rice has revealed their widespread occurrence in eukaryotes. However, little is known about *cis*-NATs in the model plant *Arabidopsis thaliana*.

**Results:**

We developed a new computational method to predict and identify *cis*-encoded NATs in *Arabidopsis *and found 1,340 potential NAT pairs. The expression of both sense and antisense transcripts of 957 NAT pairs was confirmed using *Arabidopsis *full-length cDNAs and public massively parallel signature sequencing (MPSS) data. Three known or putative *Arabidopsis *imprinted genes have *cis*-antisense transcripts. Sequences and the genomic arrangement of two *Arabidopsis *NAT pairs are conserved in rice.

**Conclusion:**

We combined information from full-length cDNAs and *Arabidopsis *genome annotation in our NAT prediction work and reported *cis*-NAT pairs that could not otherwise be identified by using one of the two datasets only. Analysis of MPSS data suggested that for most *Arabidopsis cis*-NAT pairs, there is predominant expression of one of the two transcripts in a tissue-specific manner.

## Abstract 2

A new computational method for predicting *cis*-encoded natural antisense transcripts (NATs) in *Arabidopsis* identified 1,340 potential NAT pairs. The expression of both sense and antisense transcripts of 957 NAT pairs was confirmed, and analysis of MPSS data suggested that for most pairs one of the two transcripts is predominantly expressed in a tissue-specific manner.

## Background

In the past few years, several families of regulatory RNA molecules have been shown to be widely expressed in eukaryotes [1,2]. Natural antisense transcripts (NATs) belong to one such family. NATs are endogenous RNA molecules whose partial or entire sequences exhibit complementarity to other transcripts. There are two types of NATs. *Cis*-NATs are transcribed from the same genomic loci as their sense transcripts but on the opposite DNA strand. By contrast, *trans*-NATs are expressed from genomic regions distinct from those encoding their sense transcripts [3-5]. *Cis*-NATs and their sense RNAs are usually related in a one-to-one fashion, whereas a single *trans*-NAT may target several sense transcripts; for example, one type of micro RNA (miRNA) could regulate the expression of several distinct target mRNAs [6].

Studies performed in various organisms have suggested that NATs can participate in a broad range of regulatory events, such as transcription occlusion resulting in the reciprocal expression of sense-antisense RNAs [7,8] and RNA interference (RNAi) which leads to the degradation of double-stranded sense-antisense transcript pairs [9]. There is evidence for the involvement of NATs in alternative splicing [10,11], RNA editing [12,13], DNA methylation [14,15], genomic imprinting [16-20] and X-chromosome inactivation [21]. NATs are also known to regulate expression of some circadian clock genes [22]. However, because each of the above regulatory modes was only observed in a few cases, the general biological functions and regulatory mechanisms of NATs are still unclear.

Recent large-scale NAT identifications in several model organisms have revealed the widespread existence of *cis*-NATs in eukaryotes. Lehner *et al. *first reported 372 NATs in human by searching for overlapping mRNA sequences in public databases [23]. Using a public expressed sequence tag (EST) database, Shendure and Church also found 144 human NATs and 73 mouse NATs [24]. In a later work, Yelin *et al. *predicted 2,667 NATs in human and concluded that around 1,600 NAT pairs were transcribed from both strands after experimental validation [25]. The RIKEN group identified 2,481 NAT pairs and 899 non-antisense bidirectional transcript units from 60,770 mouse full-length cDNAs [26]. A similar analysis by the same group uncovered 687 bidirectional transcript pairs from 32,127 rice (*Oryza sativa*) full-length cDNAs [27]. Antisense expression of about 7,600 annotated genes was observed in a recent work using whole-genome arrays to analyze the transcription activity of the *A. thaliana *genome. However, a detailed list of these *Arabidopsis *antisense RNAs and their complete analysis is not yet available [28]. We note that in all previous investigations NAT prediction focused on *cis*-NATs only.

Here, we present results of a genome-wide computational search to predict and identify *cis*-NATs in *Arabidopsis*. Combining sequence information of *Arabidopsis *full-length cDNAs from the public databases and *Arabidopsis *annotated genes from the *Arabidopsis *genome release, we have identified 1,340 potential *cis*-NAT pairs. Expression evidence for transcripts derived from both strands of 957 *cis*-NAT pairs was obtained from the *Arabidopsis *full-length cDNA and the public *Arabidopsis *massively parallel signature sequencing (MPSS) database.

## Results

### Prediction and identification of *Arabidopsis cis*-NAT pairs

To search for *cis*-encoded *Arabidopsis *natural antisense transcripts, we aligned all *Arabidopsis *full-length cDNA sequences collected in the UniGene and RIKEN datasets with the *Arabidopsis *genome sequences. Pairs of transcripts that satisfied the following criteria were selected as *cis*-encoded natural sense-antisense transcript pairs (referred to as NAT pairs hereafter): first, cDNAs of both transcripts can be uniquely mapped to the *Arabidopsis *genome with at least 96% sequence identity; second, the two transcripts are derived from opposite strands of the genome; third, both transcripts are encoded by overlapping genomic loci, and the overlap length is longer than 50 nucleotides; fourth, the sense and antisense transcripts have distinct splicing patterns. Applying all of the above criteria, we identified 332 sense-antisense pairs from *Arabidopsis *full-length cDNAs. These NAT pairs are referred to as cDNA-NATs.

The 332 pairs of cDNA-NATs can be grouped into two categories. The first category contained 145 NAT pairs in which both the sense and antisense transcripts had nearly perfect annotated gene matches. The second category contained 187 NAT pairs in which at least one transcript had no corresponding annotated gene. This observation led us to hypothesize that additional NAT pairs, whose corresponding cDNAs were not included in the UniGene and RIKEN *Arabidopsis *full-length cDNA datasets, could be identified using the *Arabidopsis *genome annotation.

**Figure 1:**
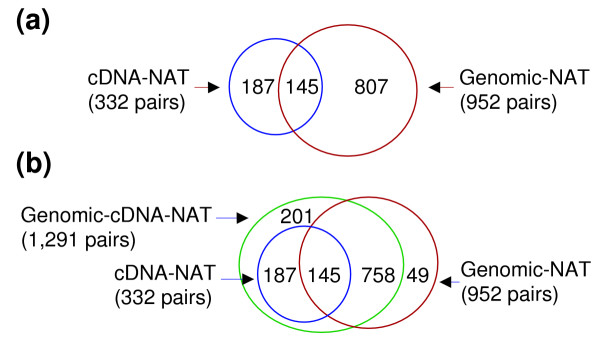
Relationships between NAT pairs from different datasets. **(a) **Overlap between cDNA-NAT pairs and genomic-NAT pairs. Among the 332 cDNA-NAT pairs, 145 pairs have corresponding annotated genes for both transcripts. For the other 187 cDNA-NAT pairs, at least one transcript has no counterpart in the current *Arabidopsis *genome annotation. **(b) **Overlap between cDNA-, genomic- and genomic-cDNA-NAT pairs. All cDNA-NAT pairs are included in genome-cDNA-NAT pairs. Blue circle, cDNA-NATs; red circle, genomic-NATs; green circle, genomic-cDNA-NATs.

To identify potential NAT pairs without full-length cDNA evidence, we compared the genomic loci of all *Arabidopsis *annotated genes to search for gene pairs that overlap in an antiparallel manner. Using the criteria described in Materials and methods, 952 putative NAT pairs were identified from the *Arabidopsis *genome and were named genomic-NATs. Among the 952 genomic-NATs, 145 pairs had corresponding full-length cDNA for both the sense and antisense genes, and therefore were also included in the cDNA-NAT set. The remaining 807 new NAT pairs were predicted using the *Arabidopsis *genome annotation only and are referred as the unique genomic-NAT set in the following analysis (Figure 1a).

For most NAT pairs in the second category of the cDNA-NAT set, only one transcript in each pair matched an annotated gene. This indicates that transcripts of some full-length cDNAs could form *cis*-NAT pairs with other transcripts, although their corresponding genes are not included in the current *Arabidopsis *genome annotation. In a search of such NAT pairs, we compared the genomic loci of the UniGene and RIKEN *Arabidopsis *full-length cDNAs with those of annotated genes and identified 1,291 full-length cDNAs whose transcripts could form *cis*-NAT pairs with potential transcripts of annotated genes (see Materials and methods for criteria). The 1,291 genomic-cDNA-NAT pairs included the 332 cDNA-NAT pairs and 758 unique genomic-NAT pairs. Therefore, 201 unique NAT pairs were predicted by the cDNA-genome comparison approach and are referred to as unique genomic-cDNA-NAT pairs hereafter (Figure 1b).

In total, we have found 1,340 potential NAT pairs from three categories: 332 pairs with cDNA evidence for both sense and antisense transcripts; 807 pairs based on the *Arabidopsis *genome annotation (including 758 pairs with full-length cDNA evidence for one strand) and another 201 genomic-cDNA pairs by combining genome annotation with full-length cDNA sequence information.

### Characterization of *Arabidopsis *NAT pairs

**Figure 2:**
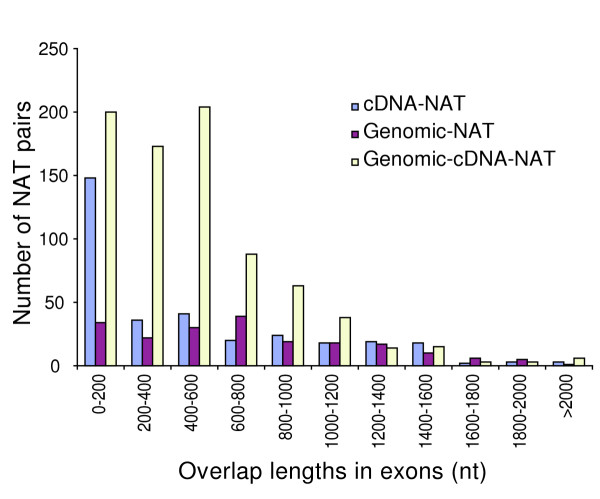
Distribution of genomic overlap lengths of NATs. The overlap length of each NAT pair in exons was calculated. The number of NAT pairs (*y*-axis) is plotted against the overlap lengths (in nucleotides) of exons in each NAT pair (*x*-axis).

We classified the 1,340 unique NAT pairs according to the exon-intron structures of each transcript and their overlapping patterns (Table 1). The overlapping patterns of NAT pairs were determined by comparing the exon positions of both transcripts using sim4 [29] alignment results. Consistent with previous reports of NAT pairs in other organisms [23-27], the majority of *Arabidopsis *NAT pairs (72.1%) overlapped at their 3' end. For almost all NAT pairs (99%), the overlapping region included exon sequences, with a few exceptions in which one transcript was transcribed entirely from the intronic sequences of the other. Figure 2 shows the distribution of overlap lengths of NATs. No obvious chromosomal bias was observed for the genomic distribution of NATs (Table 2) [30].

The sim4 cDNA alignment results showed that some *Arabidopsis *full-length cDNAs are non-spliced transcripts. To assess the quality of full-length cDNAs, we systematically compared the splicing pattern and coding potential of all full-length cDNAs used in this study to all predicted *Arabidopsis *genes. Our result showed that the proportion of non-spliced transcripts in UniGene and RIKEN full-length cDNAs was lower than the proportion of non-spliced transcripts in annotated genes, indicating non-spliced cDNAs are likely to be derived from *bona fide *transcripts rather than genomic DNA contamination (Table 3).

### Expression analysis of NAT pairs using public *Arabidopsis *MPSS data

To investigate the expression of our predicted NAT pairs, we used the public *Arabidopsis *MPSS data at the University of Delaware [31]. MPSS is a bead-based sequencing technology that identifies a sequence of 17-20 nucleotides from each transcript. This sequencing technique is capable of identifying new, rarely expressed transcripts. MPSS can also quantitatively measure the expression level of a transcript because the transcripts per million (TMP) value for a transcript in the sequencing results reflect its *in vivo *abundance [32,33].

**Figure 3:**
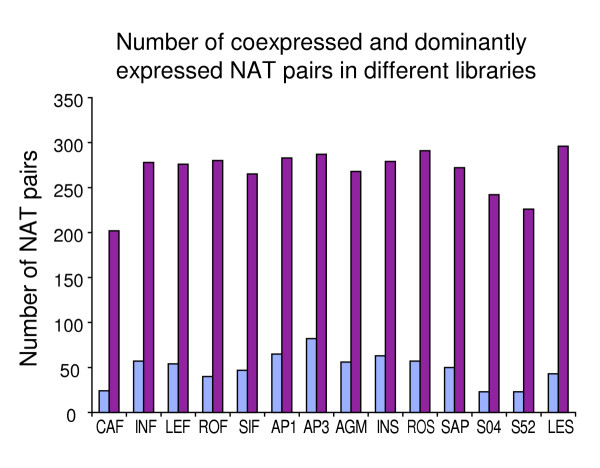
Distribution of coexpressed and dominantly expressed NAT pairs in different libraries. The number of coexpressed NAT pairs in each library was shown in blue bar and that of dominantly expressed NAT pairs in red bar. See legend of Table 5 for library information.

The public *Arabidopsis *MPSS database contains 87,705 'trusted' signature sequences from 14 cDNA libraries. By aligning these MPSS sequences to the *Arabidopsis *genome and the 1,340 NAT pairs, we identified 455 NAT pairs with unique MPSS matches on both the sense and antisense strands, including 103 cDNA-NAT pairs, 293 genomic-NAT pairs and 59 genomic-cDNA-NAT pairs. Because MPSS signatures are short 17-nucleotide sequences identified from each transcript, sequences with multiple genomic loci were excluded from our analysis to avoid ambiguity with respect to the origin of a MPSS signature and to ensure fidelity of assigning a MPSS signature to its corresponding transcript (see Materials and methods for details). Among the 455 NAT pairs with unambiguous MPSS data for both transcripts, expression of both transcripts of 78 pairs was only found in distinct libraries, indicating these NAT pairs might have an exclusive transcription relationship. For the other 377 NAT pairs, expression of the sense and antisense transcripts was mainly observed in different libraries or one transcript was dominantly expressed when both transcripts could be detected in the same library (Tables 4 and 5). For a pair of NATs found in the same library, if the TPM value of one transcript is at least three times as high as that of the other transcript, we consider that transcript as dominantly expressed. The number of coexpressed and dominantly expressed transcripts in each library was shown in Figure 3. On average, coexpression was only observed in two of the 14 tested sample libraries for each of the 377 NAT pairs, whereas dominant expression of one transcript was observed in 9 libraries. No expression was detected in the remaining libraries.

We also found additional 222 genomic-NAT pairs and 51 genomic-cDNA-NAT pairs with full-length cDNA evidence for one transcript and MPSS data for the other transcript. Together with the 332 cDNA-NAT pairs, we have obtained either full-length cDNA or MPSS expression evidence for both transcripts of 957 NAT pairs, corresponding to 71.4% of the total 1340 pairs ((455 - 103) + 332 + 222 + 51 = 957).

### siRNA matches of NAT pairs

We compared short interfering RNA (siRNA) sequences collected in the *Arabidopsis *small RNA database to investigate the possibility that *cis*-NAT pairs may generate siRNAs. Similar to the MPSS alignment process, only siRNAs with unique loci on the *Arabidopsis *genome were used in the comparison to ensure unambiguous assignment. We found 11 pairs of NATs had siRNA sequences mapped uniquely to their overlapping region (Table 6). SiRNAs of all but one NAT pairs originated from their overlap region, the only exception being pair At#S18901030 and At#S18898439, whose overlap length was only 52 nucleotides long.

### Conservation of *Arabidopsis *NAT pairs in rice

To examine whether NAT pairs might be conserved during evolution, we compared the protein sequences of the 1,340 putative *Arabidopsis *NAT pairs with the protein sequences of the 687 predicted rice NAT pairs [27]. Orthologs of two *Arabidopsis *NAT pairs were also encoded by antiparallel genes originated from the same locus in rice (Table 7). In addition, homologs of one transcript of 392 *Arabidopsis *NAT pairs were also found in the rice NAT set.

## Discussion

Although NATs are often seen in prokaryotes, their prevalence in eukaryotes was not detected until the past few years [23-27,34]. In this work, we combined sequence information on *Arabidopsis *full-length cDNAs with that from the *Arabidopsis *genome annotation and identified 1,340 potential *cis*-NAT pairs in *Arabidopsis *(Additional data file 1, 2, 3).

### Assessment of our NAT prediction methods

The 1,340 *Arabidopsis *NAT pairs were identified from three sources. First, by aligning full-length cDNA sequences to the *Arabidopsis *genome, we identified 332 cDNA-NAT pairs. However, comparison of these 332 cDNA-NAT pairs with *Arabidopsis *annotated genes showed that more than half of these NAT pairs had one partner that was not included in the current *Arabidopsis *genome annotation. Because traditional genome annotation mainly aims at the identification of protein coding genes within a genome, there is the possibility that non-coding antisense transcripts may be overlooked by currently trained gene finders. A recent report using a genome tiling array to examine the transcription activity of the entire *Arabidopsis *genome also supports this notion [28].

To search for potential NAT pairs not included in the current full-length *Arabidopsis *cDNA library, we compared the genomic coordinates of all annotated genes with each other and with those of full-length cDNAs. This approach uncovered another 807 overlapping genomic-NAT pairs based on the annotation of their corresponding genes, and 201 genomic-cDNA-NAT pairs, each including a transcript derived from an annotated gene on one strand and a transcript represented in the full-length cDNA database on the other strand. The full-length cDNAs included in genomic-cDNA-NAT pairs either had no annotated gene match or their corresponding transcripts cannot form *cis*-NAT pairs with transcripts of other genes based on their annotation. These results indicate that although the *Arabidopsis *genome is currently one of the best annotated eukaryotic genomes, a lot of information is still missing. The identification in eukaryotes of several classes of regulatory RNA genes, such as those encoding natural antisense transcripts, which are the focus here, will not only further our understanding of genome structure and gene regulation, but will also open a new window for improved genome annotation.

Most antisense prediction work reported to date has focused on identifying NATs from expressed cDNAs and ESTs [23-27]. In this work, we avoided using ESTs because of the ambiguous orientation of some sequences. We also included sequence information of annotated *Arabidopsis *genes in our NAT prediction in order to provide a more complete picture of antisense transcripts in *Arabidopsis*. The reliability of our approach is supported by the following lines of evidence: first, the expression of both sense and antisense transcripts of 293 pairs of genomic-NATs (36.3% of a total of 807) was observed in the public MPSS data, and another 222 genomic-NAT pairs (27.5% of a total of 807) have full-length cDNA evidence for one transcript and associated MPSS data for the other transcript; second, the two NAT pairs which were conserved in rice were also identified in our *Arabidopsis *genomic-NAT dataset; third, it is known that imprinted genes are likely subject to antisense regulation; three of the six reported *Arabidopsis *imprinted genes [35-39], *FIE*, *FIS2 *and *MSI1*, are included in our genomic-NAT sets. However, it remains possible that some genomic-NAT pairs are false positives if the lengths of their untranslated regions (UTRs) were annotated inaccurately.

In rice, both transcripts of 86% of the NAT pairs have coding sequence (CDS) regions whereas 28% of the predicted *Arabidopsis *NAT pairs include at least one transcript without coding potential. Non-protein-coding transcripts are more prevalent in cDNA-and genomic-cDNA-NAT pairs in that 170 cDNA NAT pairs and 156 genomic-cDNA-NAT pairs include one non-protein-coding transcript. We used Genescan to evaluate the coding potential of each transcript by screening their corresponding genomic DNA sequence for valid gene structures. Using annotated genes as controls, we estimated the false-negative rate of our definition of coding potential to be 2.3%. Unlike CDS-containing antisense transcripts that may be translated into proteins under certain conditions, transcripts without any protein-coding potential could possess solely regulatory functions.

In our work described here, and in all other genome-wide antisense transcript identification papers published so far [23-27], the investigation was focused on *cis*-antisense RNAs, which are transcribed from the same genomic loci as their sense RNAs, but on the opposite genome strand. To ensure the *cis*-antisense relationship of NATs reported here, only cDNAs with unique genomic loci were included in this study. We note that certain number of *trans*-antisense transcripts also exist in cells. Examples include miRNAs and siRNAs which are widely studied in most model organisms [6]. Genome-wide identification of *trans*-antisense transcripts in *Arabidopsis *is being attempted.

### Evaluation of NAT expression using MPSS data

The non-gel-based properties of MPSS technology render it an ideal resource for evaluating the expression profile of NAT pairs for the following reasons: first, because the MPSS technology captures almost all polyadenylated transcripts within cells, this technology is theoretically capable of identifying new, rarely expressed transcripts without prior knowledge of their corresponding genes; second, the digital result of MPSS reflects the expression pattern of a sequenced RNA molecule, and therefore provides a quantitative relationship between the sense and antisense transcript of a NAT pair in different tissues. This information was not available in any of the previous NAT prediction work [32,33].

Using the full-length cDNA and public *Arabidopsis *MPSS data, we were able to obtain expression evidence for both transcripts of 957 NAT pairs. The digital nature of MPSS data enabled us to evaluate the expression relationship of the sense and antisense transcripts directly. Our results showed that the sense and antisense transcripts of a NAT pair tend to be expressed in different tissues or under different conditions. In addition, in cases where the sense and antisense transcripts of a NAT pair were expressed in the same library, one type of transcript was usually more abundant than the other. On average, transcripts of NAT pairs were found to be coexpressed in only two libraries, whereas dominant expression (the expression level of one transcript was at least three times higher than that of the other transcript) or absolute expression (only one transcript of a NAT pair was expressed) was observed in nine libraries. The tissue-specific expression of sense and antisense transcripts observed in this study is consistent with the *Arabidopsis *genome transcription study using a whole genome-tiling array, in which about 7,600 genes were found to have tissue-specific sense and antisense expression [28]. Although a detailed list of these 7,600 genes is not yet available, it is possible that for some genes not included in our list, the antisense transcription activity was contributed by *trans*-antisense transcripts. This could explain why we predicted fewer NAT pairs than the previous work, as our work only focuses on *cis*-antisense transcripts.

To ensure the MPSS sequences were indeed generated by their matching transcripts, all MPSS data were first aligned with the *Arabidopsis *genome and all annotated mRNAs to remove signatures with multiple genomic loci. Therefore, unless an MPSS signature sequence was derived from the joint-exon region of some transcripts that are not included in the current genome annotation, it should originate from its corresponding transcript.

### Speculation on the function and origin of NATs

One possible function of NATs is to trigger the degradation of their sense transcripts via the RNAi pathway. However, in our study, we found only 11 NAT pairs with known siRNA matches. There are two possible explanations for this observation. First, the current public *Arabidopsis *siRNA database, which only contains 1,822 unique siRNA sequences, is small and does not cover all siRNAs associated with sequences of the NAT pairs reported here. Second, all NATs identified in this work are *cis*-antisense transcripts. siRNAs are used to downregulate expression levels of their target mRNA to achieve a low protein concentration. *Cis*-antisense transcripts can accomplish the same goal by interfering with the transcription of their sense transcripts, and this might be a more energy-efficient mechanism to achieve local gene regulation. This hypothesis predicts that we would expect to find more siRNAs associated with *trans*-antisense transcripts.

For most NAT pairs with associated MPSS data for both transcripts, the expression of sense and antisense transcripts tends to occur in different tissues. In these cases, we could speculate that transcription of genes encoding these NAT transcript pairs may be regulated by similar factors but that the production of antisense transcripts might interfere with the transcription of their sense transcripts, resulting in reciprocal expression patterns. Another possibility is that the two genes of a NAT pair are subject to different transcriptional regulation and consequently they are never expressed in the same tissue at the same time. Functional analysis of all NAT pairs using gene ontology reveals no over-representation of any functional category compared to the *Arabidopsis *genome, indicating that *cis*-antisense regulation might be a global mechanism for all gene families. Further experiments are needed to investigate the validity of these hypotheses.

Antiparallel transcription and antisense transcripts are known to be involved in genomic imprinting of *Xist *gene in mouse and human [21]. There is supporting evidence that the *MEA *and *PHE *genes of *Arabidopsis *are imprinted [35], and in addition, *FIS2*, *FIE*, *MSI1 *and *FWA *may also be imprinted, although the evidence for these four other genes is not unequivocal [36-39]. Nonetheless, we found antisense transcription units for *FIS2*, *FIE *and *FWA*, suggesting that transcription of these three genes might be regulated by antisense transcripts, or their antisense transcripts might be involved in silencing their expression. Genomic imprinting usually involves a chromosomal locus and, in certain cases, may even extend overa chromosomal region. Given the close proximity of the sense-antisense gene transcripts if one member of the pair is imprinted, it is likely that the other would be subject to the same regulation. Unfortunately, because of the absence of data on imprinted genes in rice, we were unable to examine whether imprinted genes were also subject to antisense regulation in rice.

We found that two *Arabidopsis *NAT pairs are conserved in rice. These conserved NAT pairs could be used to study the antisense regulatory mechanism and the origin of NATs in plants. Given over 150 million years of evolutionary distance between *Arabidopsis *and rice, the gene order on the two genomes has diverged quite significantly. Therefore, the conservation of these two NAT pairs might have some functional relevance. A closer comparison of the *Arabidopsis *and rice NAT pairs and the identification of additional conserved NAT pairs could help address this issue.

Taken together, our results provide the first genome-wide identification and prediction of NATs in *Arabidopsis*. These results will facilitate functional studies of NATs in this model plant, as well as in other plant species, and help to unravel complex gene regulatory networks in eukaryotes.

## Materials and methods

### Identification of sense-antisense transcript pairs from full-length cDNA datasets

The *Arabidopsis *UniGene (Build 45) dataset (file named At.seq.all) was downloaded from the National Center for Biotechnology Information (NCBI) UniGene Resources [40,41]. A total of 20,683 full-length cDNA sequences were extracted from the UniGene dataset by selecting sequences marked as 'Full-length/full-length cDNA'. The RIKEN *Arabidopsis *full-length cDNA dataset, which contains 13,181 sequences, was downloaded from the RIKEN BioResource Center (BRC) [42,43]. The 20,683 UniGene and 13,181 RIKEN full-length cDNAs were aligned to the *Arabidopsis *genome sequences from The Institute for Genomic Research (TIGR) (release version 5) [44] by BLAT. The splicing pattern of the transcript derived from each cDNA was further confirmed using the sim4 sequence alignment program [29,44,45]. cDNAs with at least 96% sequence identity to the *Arabidopsis *genome were used in the following analysis. For pairs of cDNAs encoded by opposite strands of the *Arabidopsis *genome and sharing overlapping genomic loci, if both their corresponding sense and antisense transcripts had no other genomic locations and exhibited different splicing patterns, they were selected as encoding sense-antisense transcript pairs and are referred to as cDNA-NAT pairs in the text.

### Prediction of sense-antisense transcript pairs using the *Arabidopsis *genome annotation and full-length cDNAs

We used the *A. thaliana *genome annotations from TIGR (release version 5) in this study [44,45]. Putative NAT pairs were identified on the basis of annotated genomic loci of *Arabidopsis *genes. If a pair of overlapping genes were located on opposite strands of the *Arabidopsis *genome and at least one gene had no annotated UTR at the overlap end, their encoded transcripts were selected as a putative NAT pair regardless of the overlap length of the encoded transcripts. Otherwise, if a pair of antiparallel overlapping genes both have annotated UTR regions at the overlap end, the overlap length of their encoded transcripts must be longer than 50 nucleotides to qualify as NAT pairs. NAT pairs from the above two categories are both referred to as genomic-NAT pairs in the text.

Genomic-cDNA-NAT pairs were identified by comparing the genomic loci of full-length cDNAs with those of annotated genes. UniGene and RIKEN full-length cDNAs with unique genomic locations and at least 96% sequence identity to the *Arabidopsis *genome were used in this step. Using the same criteria for genomic NATs, if an annotated gene had a overlap cDNA match on the opposite strand and the transcript of the annotated gene and that derived from the antisense cDNA had different splicing patterns, the gene and its matching cDNA were selected as a genomic-cDNA-NAT pair.

### Splicing pattern and coding potential evaluation of full-length cDNAs and annotated genes

Splicing patterns of transcripts encoded by full-length cDNAs were obtained by aligning the cDNA sequences to the *Arabidopsis *genome using the sim4 program [29]. Splicing patterns of transcripts derived from *Arabidopsis *annotated genes were extracted from the TIGR *Arabidopsis *genome annotation (release version 5) [44]. To evaluate the coding potential of full-length cDNAs, their corresponding genomic sequences (determined by BLAT and sim4 result) were extracted and screened by GeneScan [46].

### Identification of MPSS evidence for NAT pairs

We used the public *Arabidopsis *MPSS data at the University of Delaware [31] to evaluate the expression of NAT pairs. MPSS sequences from 14 different libraries of *Arabidopsis *Columbia-0 (Col-0) ecotype were downloaded from [31]. Each MPSS library contained signature sequences identified from the same tissue. The quality of these MPSS sequences was evaluated according to the information provided by the database. Only MPSS sequences with 'reliable' (present in more than one sequencing run) and 'significant' (TPM ≥ 4) expression pattern were considered as 'trusted' signatures and used in this analysis.

The public MPSS database contained 87,705 trusted signatures that satisfied the above expression criteria. These signatures were aligned to the sequences of the 1,340 putative NAT pairs to identify MPSS sequences derived from them. Signatures with multiple perfect matches to the *Arabidopsis *genome or to cDNAs had ambiguous origins and were not considered further. For a NAT pair, if both the sense and antisense transcripts had associated MPSS data and their expression values were both significant in one or more libraries, transcripts in this NAT pair were considered as coexpressed in the same tissue. On the other hand, if both transcripts had MPSS data but had no significant coexpression in any of the examined libraries, then the transcripts were considered as expressed, but in different libraries.

### Homology comparison with reported rice NATs

Full-length cDNA sequences of the 687 rice NAT pairs were downloaded from the website described in [27]. To facilitate protein sequence comparison, the rice and *Arabidopsis *cDNAs were mapped to their corresponding genomes by BLAT [45]. Both the *A. thaliana *and *O. sativa *genomes were downloaded from TIGR [44]. The corresponding genomic sequences of each cDNA were extracted according to their genomic coordinates from the BLAT results. Protein sequences were obtained by evaluating the genomic sequences of those cDNAs using GENSCAN [46]. The protein sequences of rice NATs were aligned with those of *Arabidopsis *NATs using blastp [47]. High similarity pairs with *E*-value less than 10^-30 ^and alignment coverage greater than 50% of query sequence were considered as homologous sequences.

## Additional data files

The following additional data are available with the online version of this paper. Additional data file 1 is a table listing all genomic-NAT pairs. Additional data file 2 is a table listing all cDNA-NATs. Additional data file 3 is a table listing all genomic-cDNA-NATs.

## Supplementary Material

Additional File 1 Classes of overlap patterns: 1. tail to tail (3' end overlap); 2. head to head (5' end overlap); 3. one transcript is contained entirely within the other transcript; 4.two transcripts overlap only within introns. Coding potential of a transcript: '+' with coding potential; '- ' without coding potential Click here for file

Additional File 2 Classes of overlap patterns: 1. tail to tail (3' end overlap); 2. head to head (5' end overlap); 3. one transcript is contained entirely within the other transcript; 4.two transcripts overlap only within introns. Coding potential of a transcript: '+' with coding potential; '- ' without coding potential Click here for file

Additional File 3 Classes of overlap patterns: 1. tail to tail (3' end overlap); 2. head to head (5' end overlap); 3. one transcript is contained entirely within the other transcript; 4.two transcripts overlap only within introns. Coding potential of a transcript: '+' with coding potential; '- ' without coding potential Click here for file

## Figures and Tables

**Table 1:** Structure analysis of NAT pairs

Category	Number of pairs			
	
	cDNA-NAT	genomic-NAT	genomic-cDNA-NAT	Total
Tail to tail (3' to 3')	181	737	48	966 (72.1%)
Head to head (5' to 5')	97	31	57	185 (13.8%)
One transcript contained entirely within the other transcript	51	35	90	176 (13.1%)
Two transcripts overlap only within introns	3	4	6	13 (1.0%)
Total	332	807	201	1,340 (100%)

**Table 2:** Chromosomal distribution of NAT pairs

Chromosome	Number of NAT pairs				Chromosome size (Mb)
		
	cDNA-NAT	genomic-NAT	genomic-cDNA-NAT	Total	
1	85	216	55	356	29.1
2	41	120	40	201	19.6
3	69	142	46	257	23.2
4	48	129	29	206	17.5
5	89	200	31	320	26.0
Total	332	807	201	1340	115.4

**Table 3:** Splicing pattern and coding potential of *Arabidopsis *full-length cDNAs and annotated genes

	UniGene cDNAs	RIKEN cDNAs	The *Arabidopsis *genome
Total transcripts	20,683	13,181	29,993
Number of transcripts with perfect genome match	17,814	12,877	29,993
Number of transcripts with ORFs	16,621	12,544	26,207
Number of non-spliced transcripts with ORFs	2,534	1,555	4,722
Number of transcripts without ORFs	1,193	333	3,786
Number of non-spliced transcripts without ORFs	466	130	3,786

The splicing pattern of each transcript was obtained by aligning its corresponding cDNA sequences to the *Arabidopsis *genome using sim4. The coding potential of the genomic sequence of each transcript was examined by GeneScan.

**Table 4:** Summary of MPSS matches for NAT pairs

	Number of NAT pairs			
	
	cDNA-NAT	genomic-NAT	genomic-cDNA-NAT	Total
Total NAT pairs	332	807	201	1,340
Number of pairs with MPSS matches on both strands				
Total	103	293	59	455
Expressed absolutely in different libraries	14	49	15	78
Expressed mainly in different libraries, occasionally in same libraries	89	244	44	377

**Table 5:** Examples of NAT pairs with MPSS matches on both strands

ID	Strand	Libraries													
		
		CAF	INF	LEF	ROF	SIF	AP1	AP3	AGM	INS	ROS	SAP	S04	S52	LES
Pair A															
At1g09750	+	*N*	*N*	*N*	*N*	*N*	**9**	*N*	*0*	*N*	*1*	*N*	*N*	*N*	*1*
At1g09760	-	*70*	*39*	*32*	*46*	*30*	**240**	*125*	*139*	*208*	*170*	*56*	*48*	*48*	*45*
Pair B															
At1g72060	+	*5*	N	*31*	2	N	N	N	N	N	2	*N*	1	*74*	*79*
At1g72070	-	*0*	N	*N*	1	N	N	N	N	N	N	*8*	N	*N*	*N*

Distinct expression of sense and antisense transcripts of NAT pair A was observed in all but one library. In the library where both transcripts of pair A were expressed, the abundance of one transcript was significantly higher than the other. For NAT pair B, the sense and antisense transcripts were expressed differentially in different libraries. Libraries in which both transcripts of a NAT pairs were expressed are shown in bold; libraries in which transcripts of only one gene of a NAT pairs were expressed are shown in italics. Abbreviations for libraries: CAF, callus - actively growing, classic MPSS; INF, infloresence - mixed stage, immature buds, classic MPSS; LEF, leaves - 21 day, untreated, classic MPSS; ROF, root - 21 day, untreated, classic MPSS; SIF, silique - 24-48 h post-fertilization, classic MPSS; AP1, ap1-10 infloresence - mixed stage, immature buds; AP3, ap3-6 infloresence - mixed stage, immature buds; AGM, agamous infloresence - mixed stage, immature buds; INS, infloresence - mixed stage, immature buds; ROS, root - 21 day, untreated; SAP, sup/ap1 infloresence - mixed stage, immature buds; S04, leaves, 4 h after salicylic acid treatment; S52, leaves, 52 h after salicylic acid treatment; LES, leaves - 21 day, untreated.

**Table 6:** siRNA matches of NAT pairs

Category of NAT pairs	Gene ID	Strand	Overlap length (nucleotides)	Description
Genomic-NAT	At2g06510	+	506	Replication protein, putative
	At2g06520	-		Membrane protein, putative
	At4g35850	+	360	Pentatricopeptide (PPR) repeat-containing protein
	At4g35860	-		Ras-related GTP-binding protein, putative
	At5g20720	+	294	Chaperonin, chloroplast
	At5g20730	-		Auxin-responsive factor
	At5g41680	+	587	Protein kinase family protein
	At5g41685	-		Mitochondrial import receptor subunit TOM7
	At5g48870	+	118	Small nuclear ribonucleoprotein, putative
	At5g48880	-		Acetyl-CoA C-acyltransferase 1
cDNA-NAT	RAFL19-56-G17	+	1,209	No coding potential
	RAFL09-70-E21	-		Expressed protein
	At#S18901030	+	52	Putative transcription factor
	At#S18898439	-		Pentatricopeptide (PPR) repeat containing protein
	At#S18900150	+	884	No coding potential
	At#S18898471	-		expressed protein
	At#S18912025	+	1,149	No coding potential
	At#S18898946	-		TCP family transcription factor
Genomic-cDNA-NAT	At1g07725	+	1,640	Exocyst subunit EXO70 family protein
	At#S18898556	-		No coding potential
	At2g16587	+	379	expressed protein
	RAFL19-48-E15	-		No coding potential

**Table 7:** Conserved NAT pairs of *Arabidopsis *and rice

		ID	Strand	Overlap pattern	Overlap length (nucleotides)	Description
NAT pair 1	*Arabidopsis*	At5g02820	+	Tail to tail	1,138	DNA topoisomerase VIA
		At5g02830	-			PPR repeat-containing protein
	Rice	J033010B03	+	Tail to tail	1	DNA topoisomerase VIA
		J013135M09	-			PPR repeat-containing protein
NAT pair 2	*Arabidopsis*	At5g54270	+	Tail to tail	1,047	Chlorophyll A-B binding protein
		At5g54280	-			Myosin heavy chain
	Rice	006-301-C08	+	Tail to tail	4,425	Chlorophyll A-B binding protein
		J013155K02	-			Myosin heavy chain
